# When condition trumps location: seed consumption by fruit-eating birds removes pathogens and predator attractants

**DOI:** 10.1111/ele.12134

**Published:** 2013-06-21

**Authors:** Evan C Fricke, Melissa J Simon, Karen M Reagan, Douglas J Levey, Jeffrey A Riffell, Tomás A Carlo, Joshua J Tewksbury

**Affiliations:** 1 Department of Biology, University of WashingtonSeattle, WA, 98195, USA; 2 Division of Environmental Biology, National Science FoundationArlington, VA, 22230, USA; 3 Biology Department, The Pennsylvania State UniversityUniversity Park, PA, 16802, USA; 4 The Luc Hoffmann Institute, WWF InternationalGland, 1196, Switzerland

**Keywords:** Endozoochory, frugivory, fungal pathogens, interaction modification, Janzen–Connell hypothesis, negative density dependence, seed dispersal

## Abstract

Seed ingestion by frugivorous vertebrates commonly benefits plants by moving seeds to locations with fewer predators and pathogens than under the parent. For plants with high local population densities, however, movement from the parent plant is unlikely to result in ‘escape’ from predators and pathogens. Changes to seed condition caused by gut passage may also provide benefits, yet are rarely evaluated as an alternative. Here, we use a common bird-dispersed chilli pepper (*Capsicum chacoense*) to conduct the first experimental comparison of escape-related benefits to condition-related benefits of animal-mediated seed dispersal. Within chilli populations, seeds dispersed far from parent plants gained no advantage from escape alone, but seed consumption by birds increased seed survival by 370% – regardless of dispersal distance – due to removal during gut passage of fungal pathogens and chemical attractants to granivores. These results call into question the pre-eminence of escape as the primary advantage of dispersal within populations and document two overlooked mechanisms by which frugivores can benefit fruiting plants.

## Introduction

Vertebrates are the dominant vectors of dispersal for virtually all the world’s most diverse plant communities, and seed dispersal by vertebrates is considered one of the key innovations in angiosperm radiation (Jordano 2000; Smith 2001; Tiffney 2004). The most commonly cited advantage of seed dispersal is deposition of seeds away from the parent plant, where the probability of survival is generally high because predators and pathogens are usually most abundant near the parent plant (Janzen 1970; Connell 1971; Bell *et al*. 2006; Mangan *et al*. 2010; Swamy *et al*. 2011). However, vertebrate consumption also alters seed condition, potentially changing seed conspicuousness, attractiveness, and vulnerability to predators and pathogens (Herrera 2002). By consuming seeds, vertebrates may thus mediate interactions with predators and pathogens via changes to both location and condition, yet most studies addressing the benefits of this mutualism for plants have focused solely on location-related benefits (Nathan & Muller-Landau 2000; Wenny 2001; Howe & Miriti 2004; Schupp *et al*. 2010).

Perhaps escape is considered the primary benefit of vertebrate seed dispersal because dispersal benefits have been most thoroughly studied in canopy trees of tropical forests (HilleRisLambers *et al*. 2002). In these species, escape from long-term, high-density seed shadows may be critical, and seed movement beyond the canopy of the parent tree typically results in seeds being deposited in environments away from conspecifics (i.e. individuals of the same species), as the tree diversity is high and the density of any one species in the community is generally low. Because the probability of being dispersed under a conspecific adult increases as the density of a species increases, rare species should benefit most often from dispersal away from the parent (Schupp 1992). For locally common species, however, escape from conspecifics is much less likely and the benefits of dispersal *per se* are presumably less important; indeed, negative density dependent survival (i.e. the decrease in survival caused by close proximity to others of the same species) is not apparent for many common tree species (Comita *et al*. 2010; Mangan *et al*. 2010; Johnson *et al*. 2012).

The reduction in escape-related benefits of dispersal should apply to any species, from trees to shrubs, as their density increases, and these benefits should disappear entirely where species become common enough that their predator and pathogen communities become functionally uniform across the landscape. For such species, condition-related benefits of gut-passed seeds may better account for advantages of vertebrate seed dispersal than might dispersal itself. To our knowledge, no previous study has experimentally compared spatial (escape) and condition-related benefits of seed consumption by frugivorous vertebrates. Doing so is important because intertwined escape-related and condition-related benefits constrain our ability to understand biological mechanisms that underlie the ecological and evolutionary interactions between fruiting plants and frugivores.

Here, we use a common understory plant species to evaluate three non-mutually exclusive hypotheses about how frugivore consumption of seeds may alter seed fate by affecting antagonistic interactions between seeds and their predators and pathogens. The ‘escape hypothesis’ posits increased seed survival resulting from the movement away from parent plants (Janzen 1970). The ‘chemical camouflage hypothesis’ posits that gut-passed seeds are either less detectable or less attractive to seed predators because gut passage removes or alters chemicals on the seed coat that would otherwise increase the risk of seed predation. Finally, the ‘pathogen removal hypothesis’ posits that passage through a vertebrate’s gut increases seed survival by removing pathogens from the seeds (Janzen 1977). The first hypothesis is a restatement of the traditional location-related benefit, whereas the second two hypotheses address condition-related benefits that are broadly applicable to all fleshy-fruited plant species, but are rarely considered as potential benefits of seed consumption (Traveset *et al*. 2007).

We test these hypotheses in *Capsicum chacoense* (Solanaceae), a species of wild chilli native to Bolivia, Paraguay and Argentina (McLeod *et al*. 1982). Fruits of this long-lived shrub are dispersed only by birds (Levey *et al*. 2006). The primary cause of seed mortality prior to dispersal is infection by *Fusarium* fungi, conveyed to seeds by fruit-piercing hemipteran insects (Tewksbury *et al*. 2008a). After dispersal has taken place, ants are the primary cause of seed mortality. To compare escape- vs. condition-related benefits of avian seed consumption, we tested three predictions, each specific to one of the three hypotheses: (1) seeds deposited far from conspecific *C. chacoense* plants will experience lower predation rates than seeds deposited near conspecifics (escape hypothesis), (2) gut-passed seeds will have a lower probability of detection by seed predators than will seeds taken directly from fruit (chemical camouflage hypothesis) and (3) seeds passed though the guts of birds will have reduced *Fusarium* fungal load and increased survival relative to unpassed seeds (pathogen removal hypothesis). To explore the mechanism for the second prediction, we also assessed whether volatile emissions from chilli seeds contain olfactory cues to seed predators.

## Material and Methods

### Species and study sites

*Capsicum chacoense* plants are long-lived and produce between 5 and 500 fruits per season from February through March; each fruit contains *c*. 18 seeds (Tewksbury *et al*. 2008b). Canopies are 0.5–1.5 m in diameter and up to 1.5 m in height. Ants were the major contributor to seed removal in exclosure experiments that isolated the impacts of insects, mammals and birds on removal of seeds (unpubl. data). Given that we frequently observed consumption of seeds in place by ants that the genera of ants that removed seeds (*Pheidole* and *Solenopsis*) are commonly considered granivorous, and that we looked for but did not find chilli seeds on ant colony refuse piles, we assume that seed removal is equivalent to seed predation. Seed germination occurs from November to January after a 5–7 month dry season. We studied two populations, one at Rancho San Julian (−19.769° −62.700°) and one at Rancho Tres Aguadas (−21.520° −63.781°) in the Gran Chaco region of southeast Bolivia.

### Escape hypothesis

Because populations of chilli plants can extend for hundreds of metres, most seeds defecated by birds likely fall within several metres of adult plants in these patches. To test the escape hypothesis within populations, we monitored predation rates of seeds placed near and far from parent plants. We used seeds passed through the gut of captive *Elaenia parvirostris*, the most common consumer of chillies at our study sites (Levey *et al*. 2006). All birds were maintained on a standardised fruit-based diet (Denslow *et al*. 1987), and all readily consumed *C. chacoense* fruits (Tewksbury *et al*. 2008b).

At both study sites, we placed groups of 10 gut-passed seeds directly on the ground at marked locations and counted remaining seeds every 48 h for 14 days. For 35 plants at each study site, we placed one group of 10 seeds under the canopy of a *C. chacoense* plant (25 cm from the plant stem; ‘near’ treatment) and a second group far from any conspecific adult (5 m from the stem of the focal plant, and > 5 m from any other chilli plant; ‘far’ treatment). At Tres Aguadas, data from day 10 were excluded from analysis because a rainstorm on day 9 washed seeds away; remaining seeds were used to assess survival rates for subsequent periods. Average between-plant distances are 2.0 m in San Julian and 0.9 m in Tres Aguadas, as estimated by 40 × 50 m stem mapping plots placed randomly within each population. Although our far treatment is only 5 m from the focal adult plant, this distance is 5 or 2.5 times greater than the average distance between the plants at San Julian and Tres Aguadas, respectively, and is thus further away from a conspecific adult than the great majority of seeds would fall.

To compare per-seed rates of daily seed predation between near and far treatments, we used a Cox regression mixed-effects model in the R package *coxme* (Therneau 2009, R Core Development Team 2012). Treatment (near or far) was included as a fixed effect, and pile ID, nested within site, was included as a random effect.

### Chemical camouflage hypothesis

To test whether gut passage reduces detectability or attractiveness of *C. chacoense* seeds by seed predators, we first conducted a field test comparing predation of gut-processed seeds vs. seeds taken directly from fruits. Next, we conducted chemical analyses of the volatiles emitted from seeds over time to determine if attractant emissions could explain differences observed in the field. Volatile analyses were conducted because seed volatiles can attract ants, and attractiveness depends in part on the concentrations of emitted volatiles (Youngsteadt *et al*. 2008, 2010; Blatrix & Mayer 2010).

For the field experiment, we established 100 plots (each 10 × 15 m) arrayed at 50-m intervals along a transect at each of the two sites. Each plot was marked with a 3 × 4 grid, thus creating 12 points, each 5 m from its nearest neighbour. We randomly chose two locations in each plot and placed 10 control seeds extracted from fruit at one, and 10 seeds passed by *E. parvirostris* at the other. Control seeds were extracted from fruit at the same time that fruits were passed by *E. parvirostris,* to control for differences in exposure time between treatments. Average seed retention time is about 40 min (Tewksbury *et al*. 2008b). We counted the number of seeds remaining in each pile every 48 h for 14 days. To compare rates of seed predation between control and gut-processed treatments, we used Cox regression mixed-effects models. Treatment (gut-processed or control) was the fixed effect, and pile ID, nested within site, was the random effect.

To identify *C. chacoense* seed volatiles and determine their emission rate over time, we used solid phase microextraction fibres (SPME) (Pawliszyn 1998; Vas & Vekey 2004). Seeds were collected from ripe fruits harvested from *C. chacoense* plants grown in a glasshouse. We divided the seeds into three groups of six samples, each containing 10 seeds. Volatiles were collected on the first, second and fourth days after seeds were removed, corresponding to days 0, 1 and 3 of the field experiments. Seeds were kept in a chamber with a 31°C (day): 21°C (night) cycle prior to sampling to replicate field temperature conditions.

Samples were placed in 10-mL glass screw vials with ultraclean screwcaps with teflon septa for SPME sampling (Agilent Technologies, Palo Alto, CA, USA). After 1 h equilibration, a SPME fibre (black; 75 μm Carboxen-PDMS; Supelco Analytical, Bellefonte, PA, USA) was inserted into the vial and exposed for 1 h. The adsorbed volatiles were injected to a gas chromatograph–mass spectrometer (GCMS) by desorption at 200 °C for 2 min in the injector (splitless mode). The GCMS analysis was done on HP 7890A GC and a 5975C Network Mass Selective Detector (Agilent Technologies, Palo Alto, CA, USA). A DB1 GC column (J &W Scientific, Folsom, CA, USA; 30 m, 0.25 mm, 0.25 μm) was used, with helium as carrier gas at constant flow of 1 cc min^−1^. The initial oven temperature was 50 °C for 4 min, followed by a heating gradient of 10 °C min^−1^ to 250 °C, which was held isothermally for 10 min. Chromatogram peaks were identified tentatively with the aid of the NIST mass spectral library (ca. 120 000 spectra) and verified by chromatography with authentic standards (when available). Peak areas for each compound were integrated using ChemStation software (Agilent Technologies, Palo Alto, CA, USA) and are presented in terms of nanograms per 10-seed sample per hour.

### Pathogen removal hypothesis

We used two experiments to assess how gut passage affects fungal pathogen load and seed survival. The first isolated the mechanism under laboratory conditions and tested whether gut passage alters pathogen load. The second determined the consequences of gut passage on seed survival under natural conditions when post-dispersal seed predators are excluded.

The first experiment used the seed infection scoring system developed by Tewksbury *et al*. (2008a) to compare severity of fungal infection for gut-processed seeds vs. those removed directly from fruit. Fungus is typically present at the time of dispersal, but grows, becomes visually apparent, and can be scored several months after dispersal, but before germination. Each seed received a score of 0–5 on each side of the flat seeds. A score of zero represents no sign of fungal infection and a score of five represents complete coverage of fungus on the seed; scores were summed across the two sides for each seed to yield a score between 0 and 10. Seed survival probability is negatively related to fungal infection score (Tewksbury *et al*. 2008a). To obtain paired gut-processed seeds and unprocessed seeds, we first harvested ripe *C. chacoense* fruits from local plants. We removed 3–5 seeds directly from each fruit through a small incision. We then presented the remainder of the fruit (typically containing 13–15 seeds) to one of nine randomly assigned captive *E. parvirostris* (see Tewksbury *et al*. 2008b for feeding protocols). We retrieved passed seeds from faeces, stored all seeds outside in a cage that allowed air flow, but blocked rain and pests, and scored seeds after 3 months. We compared fungal infection on unprocessed and gut-processed seeds from 12 trials with a paired *t*-test, which controls for individual differences among fruits.

In the second experiment, we assessed the consequences of gut passage on seed survival in the field over the 5–6-month dry season, between dispersal and germination. We collected fruits from local chilli plants, fed half of them to captive *E. parvirostris*, and removed seeds directly from the remaining fruit. We then placed seeds, in groups of 5, in small (30 mm diameter) plastic cups filled with local soil with drainage holes in the bottom. Individual cups contained seeds of one of the two treatments, and in total, 108 cups had gut-processed seeds and 108 cups had seeds taken directly from fruit (*n* = 1080 seeds in total). To exclude seed predators, and thereby isolate impacts of fungal pathogens, all cups were covered with fine nylon mesh, secured with a rubber band and buried so that the lip of the cup was *c*. 2 mm above the soil surface. Cups were placed in the field during the fruiting season in March and retrieved after the end of the dry season. When cups were removed, germinated seeds were easily recognised due to the persistence of the hypocotyl and the presence of the open seed coat, which was often found attached to the tip of the hypocotyl. Ungerminated seeds were cut in half and stained with tetrazolium chloride to determine viability (Cottrell 1947). Survival data of seeds were analysed using general linear mixed models with a binomial error distribution in the R package *lme4* (Bates *et al*. 2011). Treatment (gut-processed or control) was the fixed effect, and cup ID and plot ID were random effects; time in the field (number of days) was included as a covariate. We were unable to test both the impact of gut passage on fungal load and the impact of gut passage on field survival in the same experiment because discoloration from soil on seeds in the cups precluded accurate fungal scoring.

## Results

### Escape

**Figure 1:**
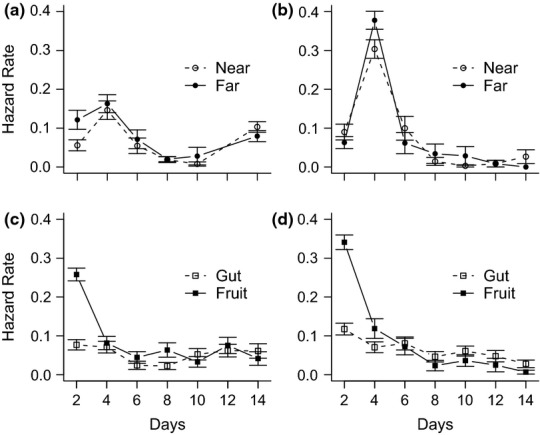
Impact of distance and gut passage on hazard rate (daily per-seed probability of predation) at Rancho San Julian (a,c) and Rancho Tres Aguadas (b,d) study sites. Escape Hypothesis (a–b): predation rates were greater for seeds placed 5 m from any *Capsicum chacoense* plant (‘Far’) than for seeds placed under the canopy (‘Near’) (Cox regression, *P* = 0.028), contrary to the escape hypotheses. Chemical Camouflage Hypothesis (c–d): seeds taken directly from fruit (‘Fruit’) suffered greater predation rates than gut-passed seeds (‘Gut’) (Cox regression, *P* = 0.00001), particularly in the first 2 days of the experiment. Error bars indicate +/− 1 SE.

Removal rates of seeds placed far from adult plants were higher than removal rates of seeds placed under adult plants (Cox regression, *P* = 0.028; Table 1), providing no support for the escape hypotheses at the scale of the populations tested. By the end of 14 days, 72% of seeds placed near adult plants and 79% of seeds far from adult plants were removed. Although sites differed in per-seed daily predation rates, these rates peaked in the first days of the experiment; the magnitude of the difference between near and far was small and relatively constant over time (Fig. 1a–b).

**Table 1:** Analysis of distance (escape hypothesis) and gut passage (chemical camouflage hypothesis) on seed survival using Cox proportional hazard models with mixed effects

Hypothesis	Parameter	Coefficient	SE	*z*	*P*
Escape	Distance	0.295	0.134	2.2	0.028
Camouflage	Gut passage	−1.356	0.130	−10.4	<0.00001

### Chemical camouflage

Seeds passed through the guts of birds were much less likely to be removed than were seeds taken directly out of fruits (55% vs. 78% after 14 days; Table 1; Cox regression, *P* < 0.00001). This doubling of survival was generated by differences in removal rates during the first 2 days of the experiment (Fig. 1c–d), indicating that seeds not gut-passed were more detectable or attractive. Although the effect was transient (i.e. rates of per-seed survival between treatments did not differ after the second day), the difference it generated in survival of passed and non-passed seeds persisted for the duration of the experiment (Fig. 1c–d). This effect may be caused by gut passage removing volatile compounds of seeds that attract predators. Indeed, our collection of volatile compounds under laboratory conditions revealed a 100-fold decrease in emission rates from the first to third day post-removal (496 ng h^−1^ vs. 4 ng h^−1^; *t*-test: *t* = 2.36, *P* < 0.05), corresponding to the time when seeds in both treatments began experiencing similar predation rates. The volatile chemicals from unprocessed seeds included aromatic and aliphatic compounds that are known attractants to *Pheidole* and *Solenopsis* ants (Figure S1, Table S1).

### Pathogen removal

**Figure 2:**
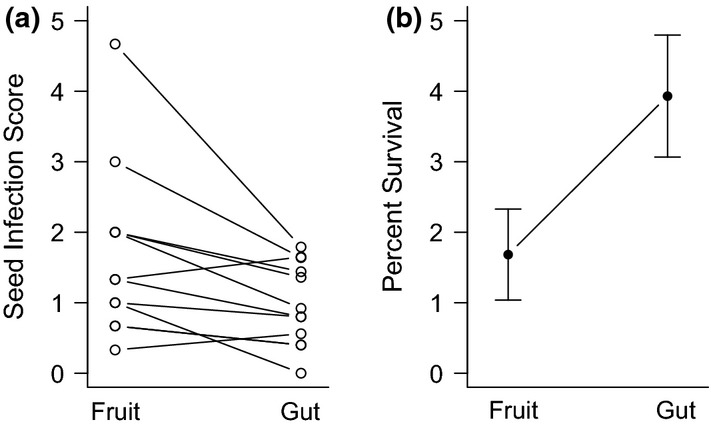
Effect of gut passage on seed infection score and survival in the field. (a) Seeds from 12 *C. chacoense* fruits were taken directly from fruit (‘Fruit’) or passed though the gut of birds (‘Gut’). Gut passage reduces fungal load of seeds relative to control seeds taken directly from fruit (mean effect size = 0.313; paired *t* = 2.79, d.f. = 11, *P* = 0.018). (b) Survival through the dry season for gut-passed and unprocessed seeds. Gut passage increased probability of survival (likelihood ratio test, χ^2^ = 3.86, *P* = 0.049). Error bars indicate ± 1 SE.

In the first experiment, which used seeds stored in sheltered conditions for 3 months, gut passage reduced fungal load on seeds by 31% relative to control seeds taken from the same fruit (paired *t* = 2.79, d.f. = 11, *P* = 0.018; Fig. 2a). In the second experiment, which used seeds placed on soil in natural conditions for 6 months, gut-passed seeds had twice the survival of control seeds taken directly from fruits (likelihood ratio test, χ^2^ = 3.86, *P* = 0.049; Fig. 2b); 3.9% of gut-passed seeds survived, whereas only 1.7% of control seeds survived. These results indicate that seed ingestion reduces *Fusarium* load and that this reduction doubles seed survival in nature.

## Discussion

We assessed the mechanism and consequence of both escape- and condition-related benefits of seed consumption by frugivores, and found that the benefits derived from changes to seed condition in *C. chacoense* outweigh the traditionally oriented benefits of dispersal within populations. Fruit consumption greatly reduced seed predation by removing volatile chemicals known to attract granivorous ants, and decreased seed mortality due to pathogens (Tewksbury *et al*. 2008a) by reducing fungal pathogen load. Together, these condition-related benefits of gut passage increased seed survival by 370%. Whereas changes in seed location are broadly recognised to affect species interactions, studies examining the effects of changes in seed condition typically focus on intrinsic characteristics such as germinability (Traveset *et al*. 2007; Schupp *et al*. 2010). We show strong experimental support that changes to condition alter species interactions.

Because our study focused on dispersal within established populations of *C. chacoense*, we cannot assess the relative importance of escape-related benefits that might apply to dispersal at larger scales (Nathan 2006). As in most studies of dispersal benefits, we did not quantify benefits of escape that may apply outside of populations, or the fraction of seeds to which such benefits might apply. Still, we are able to conclude that distances that are relatively far from conspecifics within populations – several times greater than mean between-plant distances – are not sufficient for location-related benefits in this species. Research to assess the distance necessary to gain location-related benefits, and how these distances vary with species abundance, would provide greater insight into the nature of dispersal benefits for plants species of differing local abundance.

The interaction modifications caused by frugivory alone can produce strong benefits of the plant-frugivore mutualism, and condition-related advantages of frugivory are likely common in seed dispersal mutualisms. For example, other studies have reported benefits of pulp removal, even when seeds are not consumed or dispersed (Meyer & Witmer 1998; Lambert 2001; Fedriani *et al*. 2012). Condition-related benefits may be particularly important in common species, for which the traditional escape-related benefits of animal-mediated seed dispersal may be small, or may apply to only a very small fraction of seeds (Comita *et al*. 2010; Mangan *et al*. 2010; Johnson *et al*. 2012). Consumption effects on seed condition have the potential to change many post-dispersal interactions, and the lack of strong escape-related benefits of seed consumption does not necessarily indicate that the mutualism between these fruiting plants and their fruit consumers is any less important for these species. Even without escape-related benefits in *C. chacoense*, consumption of seeds by frugivorous birds resulted in large reductions in interactions with predators and pathogens, and the mechanisms were entirely due to changes in seed condition.

Consumption of seeds may reduce interactions with predators and pathogens by several mechanisms. Our results support the hypothesis that gut passage removes pathogens on seeds (Janzen 1977). Although, the effects of gut passage on intrinsic physical seed properties that influence germinability and the effects of gut passage on species interaction, like in many studies, cannot be distinguished, reduction in fungi on *C. chaocense* seeds is likely important because fungal pathogens are a major cause of mortality (Tewksbury *et al*. 2008a). Furthermore, impacts of gut passage on intrinsic physical seed properties are unlikely to increase survival because greater physical breakdown of the seed coat during gut passage reduces survival in this species (Tewksbury *et al*. 2008b). More generally, studies that test for impacts of scarification using gut-passed and unpassed seeds may inadvertently quantify benefits of pathogen removal. We also provide evidence that seed passage alters cues used by granivorous ants. In particular, *C. chacoense* seeds taken directly from fruits emit alkaloid volatiles that also serve as olfactory cues for ants (Table S1; Vander Meer *et al*. 2010; Sonnet 1972; Morgan 2009).

These findings of strong, spatially independent benefits of animal-mediated seed dispersal have implications for the role of dispersal in the maintenance of plant species diversity. Many authors have argued that natural enemies promote diversity through conspecific negative density dependence (Adler & Muller-Landau 2005; Freckleton & Lewis 2006; Terborgh 2012). To test for density dependence, it is typical to compare rates of mortality, predation or infection as a function of distance from a parent tree or of conspecific density. Greater per-seed rates of mortality at locations near conspecific trees or in locations with high seed density are considered evidence for advantage-when-rare benefits caused by negative density dependence.

We suggest this approach overestimates the advantage-when-rare benefits of escape. Seed condition varies systematically between areas near and far from conspecifics (or areas of high and low seed density); seeds that fall near parent trees (or in areas of high conspecific seed density) are less likely to have been dispersed by a vertebrate. In contrast, for most animal-dispersed species, seeds falling far from parent trees are almost always processed by vertebrates before arrival. Our results indicate that greater survival far from parent plants is likely due at least in part to density-independent benefits of gut processing rather than negative density dependence. Indeed, if our study had ignored seed condition and half of our ‘near’ seeds were not gut-passed, while ‘far’ seeds were all gut-passed (a situation that is common in many tropical species) movement away from the parent plant would appear to nearly double the chance of survival. Such a result would be interpreted as strong support for the advantage-when-rare benefits of escape. However, density-independent benefits of gut passage actually account for all of these differences. Changes to condition provide a second axis along which impacts of dispersal should be considered.
