# Supplementary figures and images for: When condition trumps location: seed consumption by fruit-eating birds removes pathogens and predator attractants
Source: Ecol Lett. 2013 Jun 21;16(8):1031–6. doi: 10.1111/ele.12134 (PMC3806274; doi:10.1111/ele.12134)

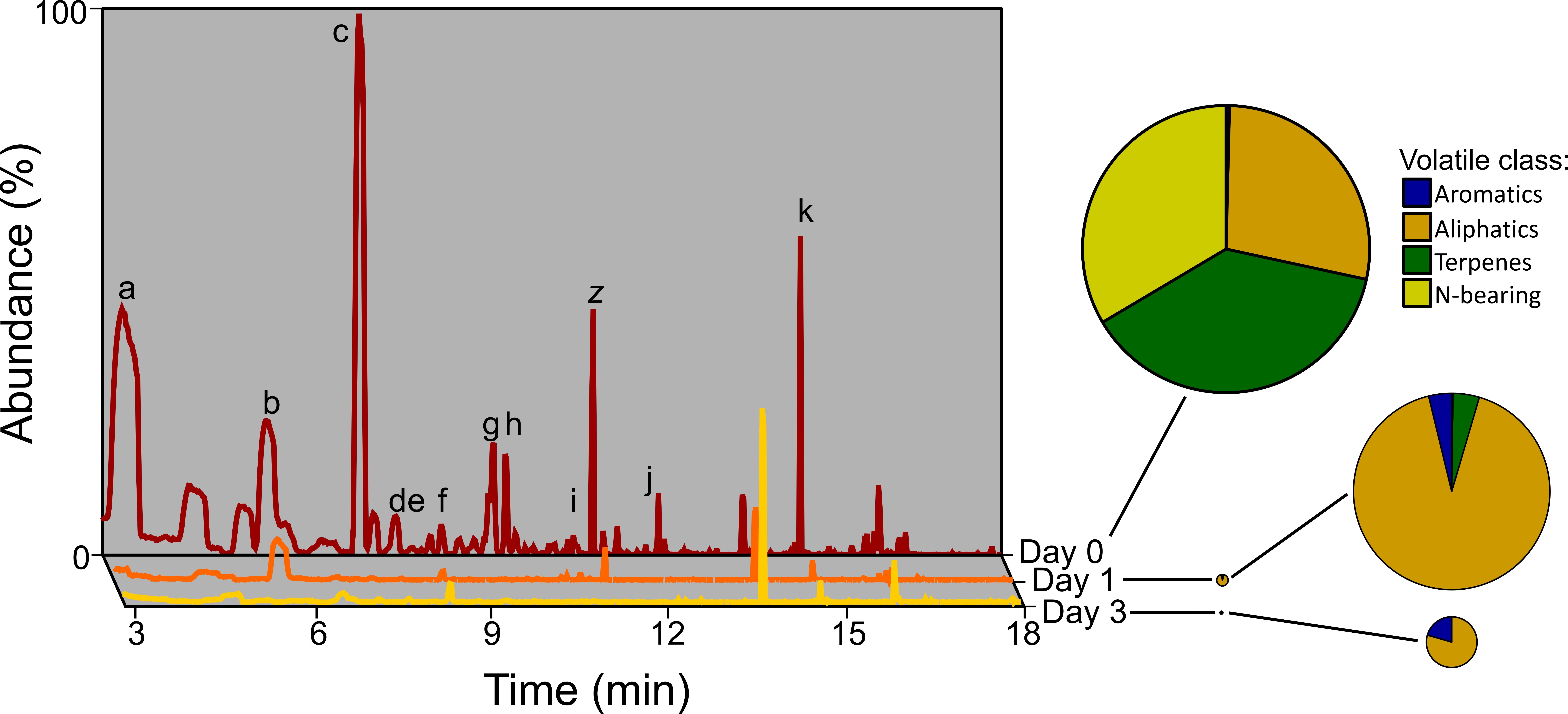

Supplement: Supplementary file 1 [file ele0016-1031-sd1.jpg]
