# Supplementary material for: When condition trumps location: seed consumption by fruit-eating birds removes pathogens and predator attractants
Source: Ecol Lett. 2013 Jun 21;16(8):1031–6. doi: 10.1111/ele.12134 (PMC3806274; doi:10.1111/ele.12134)
Supplement: Supplementary file 2 [file ele0016-1031-sd2.docx]

**Table S1.** GCMS analysis of the *Capsicum chacoense* seed volatiles over the course of a four-day time period (N=6 replicates of 10 seeds for each time period).

|  | Treatment | | |
| --- | --- | --- | --- |
|  | Day 0 | Day 1 | Day 3 |
|  |  |  |  |
| Emission rate (ng/h)* | 496.0 (207.6) | 19.8 (4.4) | 4.2 (0.8) |
|  |  |  |  |
| Compound (ng/h) |  |  |  |
|  |  |  |  |
| **1-methylpyrrole** | 161.2 (67.5) | nd | nd |
| **hexanal** | 35.6 (14.9) | 2.8 (0.6) | nd |
| **2-hexenal** | 52.0 (21.8) | 14.8 (3.3) | nd |
| tricyclene | 134.4 (56.2) | 0.2 (0.1) | tr |
| **camphene** | 8.5 (3.5) | nd | nd |
| **2-methoxy-3-**  **methylpyrazine** | 1.78 (0.7) | nd | nd |
| ***β*-thujene** | 0.3 (0.1) | nd | nd |
| ***β*-myrcene** | 4.0 (1.6) | nd | nd |
| **eucalyptol** | 20.4 (8.5) | 0.2 (0.1) | nd |
| ***cis-β-*ocimene** | 9.5 (4.0) | nd | nd |
| **tetramethylpyrazine** | 0.8 (0.3) | nd | nd |
| **2-isobutyl-3-**  **methoxypyrazine** | 0.2 (0.1) | nd | nd |
| **tetradecane** | 0.4 (0.2) | tr | nd |
|  |  |  |  |

**t*-test: *P*<0.05

Compounds in bold are those verified by synthetic standards

nd. denotes volatile not detected by the MS

tr. denotes trace levels of the volatile
