# Supplementary material for: When condition trumps location: seed consumption by fruit-eating birds removes pathogens and predator attractants
Source: Ecol Lett. 2013 Jun 21;16(8):1031–6. doi: 10.1111/ele.12134 (PMC3806274; doi:10.1111/ele.12134)
Supplement: Supplementary file 3 [file ele0016-1031-sd3.docx]

SUPPORTING INFORMATION

**Figure S1.** GCMS analysis of the headspace volatiles from chili seeds. Constituents of chili seed headspace shown in the total ion chromatogram for day 1 (red trace), 2 (orange trace), and 4 (yellow trace). Individual constituents include nitrogen-bearing alkaloids: 1-methyl pyrrole (a), 2-methoxy-3-pyrazine (e), tetramethylpyrazine (i), and 2-isobutyl-3-methoxypyrazine (j); monoterpenes tricyclene (c), camphene (d), *β*-myrcene (f), eucalyptol (g), and *cis-β*-ocimene (h); and aliphatics 2-hexenal (b), and 2-methyl-tridecane (j). *z* denotes a contaminant. Over the course of 4 d total emission rates decreased from 496 ng/h (± 207 ng/h SEM) to 4.2 ng/h (± 0.7 ng/h SEM). In addition, the composition of the headspace volatiles changed over the course of the experiment, with the composition being dominated by nitrogen-bearing pyrazines and pyrroles – known pheromone attractants of ants – at day 0, to the composition dominated by aliphatics at day 3. Pie charts to the right of the chromatogram are the absolute amounts on the same scale, and pie charts to the far right are those for day 1 and 3 at an increased scale but relative to one another. Constituents are classified by chemical class: aromatics, aliphatics, terpenes, and nitrogen-bearing (N-bearing).
